# Complete Genome Sequence of Lactobacillus crispatus CO3MRSI1

**DOI:** 10.1128/MRA.01538-18

**Published:** 2019-01-31

**Authors:** Elias McComb, Johanna Holm, Bing Ma, Jacques Ravel

**Affiliations:** a Institute for Genome Sciences, University of Maryland Baltimore, Baltimore, Maryland, USA; Georgia Institute of Technology

## Abstract

Lactobacillus crispatus is a commonly found bacterium in vertebrate microbiota, particularly the human vagina. We report the first complete genome of a strain isolated from a human vagina, L. crispatus CO3MRSI1.

## ANNOUNCEMENT

Lactobacillus crispatus is frequently associated with vertebrate microbiota, particularly the human vaginal microenvironment (1). To date, the only closed genome to represent the species has been a strain isolated from a chicken crop (2). We report the first completed Lactobacillus crispatus genome sequence of a strain isolated from the human vagina. L. crispatus has often been associated with beneficial health outcomes such as decreased risk for sexually transmitted infection and lowered prevalence of pregnancy complications (3, 4). This genome will serve as a reference for studies aiming to better understand human-associated strains of L. crispatus and their roles in reproductive health.

L. crispatus CO3MRSI1 was isolated from a midvaginal swab collected from a participant in a study in Baltimore, MD, in 2010 (1). The University of Maryland Baltimore (UMB) institutional review board (IRB) approved the use of this sample. The swab was stored frozen in Amies transport medium, and then 50 µl of thawed transport medium was plated onto a de Man-Rogosa-Sharpe (MRS) agar plate (Sigma-Aldrich, Carlsbad, CA) that was grown under anaerobic conditions in a Coy chamber (Grass Lakes, MI) at 37°C. After 48 h, a colony was picked and grown in MRS broth under the same conditions for 48 h. The DNA was extracted by removing the S-layer proteins (described by Johnson et al.) (5), followed by extraction using the MasterPure DNA purification kit (Lucigen, product no. MC85200) with two phenol-chloroform cleanups prior to DNA precipitation. The genomic DNA was sheared to 20 kb using needle shearing and was size selected using BluePippin (Sage Scientific, Beverly, MA). SMRTbell libraries were generated following standard library protocols of a DNA template preparation kit (Pacific Biosciences, Menlo Park, CA) and sequenced using one single-molecule real-time (SMRT) cell on a PacBio RS II system with the mode set at 7,750 bp. Library construction and sequencing were performed at the Institute for Genome Sciences at UMB.

Raw sequence reads (197,711 reads) were quality filtered and removed if they were less than 1,000 bp long and had less than 5% overlap. The average read length was 10,952 bp, resulting in 33.1× coverage of the genome. Reads were assembled using Canu v1.7 with default settings (6). The strain was identified as L. crispatus by means of phylogenetic analysis of both the full-length 16S rRNA gene sequence (99% sequence identity with the 16S rRNA genes in L. crispatus DSM 20584 and L. crispatus ATCC 33820) and marker genes using an adaptation of FastTree2 in Kbase (SpeciesTreeBuilder v0.0.12) (7). L. crispatus CO3MRSI1 consists of a single circular chromosome (2,345,902 bp) with a GC content of 37.1%. No plasmid was detected. Gene finding and annotation were performed on Kbase using Prokka v1.12 (8). The assembly contains 2,307 predicted coding sequences (CDS), 55% with putative function. The genome contains 5 rRNA operons and 64 tRNA genes (9). A single clustered regularly interspaced short palindromic repeat (CRISPR) locus was detected using CASC v2.5 (10).

### Data availability.

The whole-genome sequence of L. crispatus CO3MRSI1 has been deposited in GenBank under the accession no. CP033426. Raw sequencing reads of the genome of L. crispatus CO3MRSI1 are available in the SRA under the accession no. PRJNA499123. Marker gene phylogeny and gene annotation were deposited in Kbase (https://narrative.kbase.us/narrative/ws.37413.obj.19) for public assessment.
